# Cost-effectiveness analysis of camrelizumab plus chemotherapy as first-line treatment for advanced squamous NSCLC in China

**DOI:** 10.3389/fpubh.2022.912921

**Published:** 2022-08-15

**Authors:** Taihang Shao, Yinan Ren, Mingye Zhao, Wenxi Tang

**Affiliations:** ^1^Center for Pharmacoeconomics and Outcomes Research, China Pharmaceutical University, Nanjing, China; ^2^Department of Public Affairs Management, School of International Pharmaceutical Business, China Pharmaceutical University, Nanjing, China

**Keywords:** cost-effectiveness analysis, camrelizumab, advanced squamous NSCLC, CameL-sq, China

## Abstract

**Objective:**

Results of CameL-sq has revealed the clinical benefits to patients with advanced squamous non-small-cell lung cancer (sq-NSCLC). This study aims to evaluate the cost-effectiveness of camrelizumab plus chemotherapy to treat sq-NSCLC from the perspective of the Chinese healthcare system.

**Methods:**

We used a partitioned survival model with a lifetime horizon to evaluate the cost-effectiveness of camrelizumab plus chemotherapy vs. chemotherapy in treating sq-NSCLC. Baseline characteristics of patients and key clinical data were extracted from CameL-sq. Costs and utilities were collected from the open-access database and published literature. Costs, quality-adjusted life-years (QALYs), life-years gained, and incremental cost-effectiveness ratios (ICERs) were chosen as economic outcome indicators. We also performed a sensitivity analysis, subgroup analysis, and scenario analysis to verify the stability of the basic analysis results and explore the results under different scenarios.

**Results:**

Combination therapy added 0.47 QALYS and 0.91 life-years with an incremental cost of $6,347.81 compared with chemotherapy, which had an ICER of $13,572 per QALY. The probabilistic sensitivity analysis indicated that camrelizumab plus chemotherapy had a 37.8% probability of cost-effectiveness at a willingness-to-pay threshold (WTP) of 1 time GDP per capital. When WTP was set as 3 times GDP per capital, combination therapy had significant cost-effectiveness. Deterministic sensitivity analysis showed that cost of the best supportive care was the factor with the greatest influence. The subgroup analysis found that combination therapy was associated with cost-effectiveness in several subgroups, namely, patients with disease stage IIIB/IIIC and with PD-L1 tumor proportion score ≤ 1%. Scenario analysis showed that ICER was positively correlated with the price of camrelizumab.

**Conclusion:**

In this economic evaluation, camrelizumab plus chemotherapy was unlikely to be cost-effective compared with chemotherapy in the first line therapy of sq-NSCLC from a perspective of the Chinese healthcare system. Reducing the price of camrelizumab and tailoring treatments based on individual patient factors might improve the cost-effectiveness. Our findings may provide evidence for clinicians in making optimal decisions in general clinical practice.

## Introduction

With an estimated 2.1 million new cases and a cancer-related mortality of 18.4% in 2018, lung cancer remains the leading common malignancy worldwide (1). Non-small cell lung cancer (NSCLC) accounts for approximately 83% of all the lung cancers (2), of which the squamous histological subtype accounts for nearly 30% (3). Treatment for squamous NSCLC (sq-NSCLC) is a challenge because of its specific clinicopathologic features (4). The standard first-line treatment for advanced sq-NSCLC has been platinum-based doublet chemotherapy for decades with a median overall survival (OS) of 8.1 to 10.3 months (5). And many currently available treatment options developed for NSCLC are not approved or are not suitable for use in patients with the squamous histological subtype (6).

The emergence of programmed death-(ligand) 1 (PD-(L)1) inhibitors has drastically altered the landscape of the treatment of sq-NSCLC. Several studies have reported the efficacy of using the combination of PD-(L)1 inhibitors and chemotherapy. KEYNOTE-407 and Impower-131 which both targeted the global populations reported substantial improved progression-free survival (PFS) with the pembrolizumab or atezolizumab plus platinum-based chemotherapy (7, 8). RATIONALE-307, ORIENT-12, and Gemstone-302 indicating that the combination therapy (tislelizumab, sintilimab, or sugemalimab plus chemotherapy) both significantly prolonged survival years in the Chinese patients (9–11).

Camrelizumab (SHR-1210) is a newly developed monoclonal antibody against PD-1, which has shown good clinical benefit in many other tumor types, namely, hepatocellular carcinoma, esophageal squamous cell carcinoma, and non-sq-NSCLC (12–14). Recently, the CameL-sq (NCT03668496), a randomized phase 3 trial evaluated the combination of camrelizumab and carboplatin plus paclitaxel in the Chinese patients with sq-NSCLC (15). CameL-sq revealed that the camrelizumab combined with chemotherapy significantly prolonged PFS (median, 8.5 vs. 4.9 months) and OS (median, not reached vs. 14.5 months), with no unexpected treatment immune-related adverse events (15). In addition, the price of camrelizumab per cycle in the National Reimbursement Drug List (NRDL) is $460.31. Comprehensively considering the clinical benefits and potential cost-effectiveness, the Chinese government approved this combination therapy for the first-line treatment of sq-NSCLC in 2021.

In spite of these encouraging clinical results, evidence of cost-effectiveness should not be ignored since the combination therapy had a relatively higher cost when compared with chemotherapy alone. Therefore, the aim of this cost-effectiveness analysis was to compare camrelizumab plus chemotherapy with chemotherapy alone in advanced sq-NSCLC from the perspective of the Chinese healthcare system. We also aimed to analyze its cost-effectiveness under different scenarios to provide evidence for Chinese governments, clinicians, and patients.

## Materials and methods

### Study overview

This study followed the Consolidated Health Economic Evaluation Reporting Standards (CHEERSs) reporting guideline (16). Targeted patients were Chinese adults (aged ≥ 18 years) who had pathologically confirmed stage IIIB-IV sq-NSCLC, had not previously received systemic therapy, which was the same as that from CameL-sq trial. Included patients received camrelizumab (200 mg) or placebo combined with carboplatin (area under the curve 5 mg/ml per min) plus paclitaxel (175 mg/m^2^) for 4 to 6 cycles, followed by maintenance therapy with camrelizumab or placebo (15). Body surface area was assumed to be 1.72 m^2^ and creatinine clearance was assumed to be 70 ml/min (17, 18). The first-line treatment were discontinued when disease progressed and patients in both arms could receive second-line treatment. In addition, patients in chemotherapy group could cross over to immunotherapy with BICR-assessed (Blinded Independent Central Review) disease progression. In CameL-sq trial, 47% of disease progressed patients in chemotherapy group crossed over to use camrelizumab. We supposed that second-line treatment included the best supportive care and chemotherapy (mainly docetaxel) (19).

### Model construction

We developed a partitioned survival model to compare healthcare costs and clinical outcomes associated with camrelizumab plus chemotherapy vs. chemotherapy for treatment of patients with advanced sq-NSCLC (20). This model containing 3 mutually exclusive health states: progression-free survival (PFS), progressed disease (PD), and death. The time horizon was 8 years with more than 99% patients died in both treatment arms, that is, we considered a lifetime horizon (21, 22). The cycle length was a treatment cycle (21 days). Since we aimed to provide the evidence of the resource costed from using camrelizumab and the related benefits brought to patients, we selected to conduct this analysis from the perspective of Chinese healthcare system. The primary output of the model were life-years, quality-adjusted life-years (QALYs) and incremental cost-effectiveness ratio (ICER). Both costs and utilities were discounted by 5% annually (23). A willingness-to-pay (WTP) threshold was set as $12,728 (1 time GDP per capita) per QALY. We also explored the cost-effectiveness by ranging the threshold from $12,728 to $38,184 per QALY gained (3 times GDP per capita) (24). The model was constructed using R 4.1.2 (https://www.r-project.org/) and Microsoft Excel (Redmond, Washington, United States). We used R packages “flexsurv” and “survHE” to reconstruct IPD and extrapolate survival outcomes.

### Effectiveness

Probabilities of OS and PFS were extracted from the Kaplan–Meier (KM) curves in the CameL-sq using GetData Graph Digitizer (http://getdata-graph-digitizer.com) followed the method of Guyot to reconstruct estimates of individual patient data (IPD) over the clinical trial time (15, 25). Virtual IPD comprised event and censor times and were almost equal in number to the initial number at risk, which closely reproduced the digitized KM curves. These data points were then used to fit the following parametric functions: exponential, weibull, gompertz, gamma, log-logistic, log-normal, generalized gamma, genf, fractional polynomial (FP), restricted cubic spline models (RCS), and Royston–Parmar (RP) spline models. Details of methodology are shown in the Supplementary material. Goodness-of-fit was evaluated through visual inspection and the Akaike information criterion (AIC) (26). Lower AIC values combined with reasonable visual effects indicate a better fit of the selected model (27). The parameters of final survival functions of the camrelizumab plus chemotherapy and chemotherapy are shown in Table 1, goodness-of-fit are shown in the Supplementary material.

**Table 1:** Key model inputs.

**Parameters**	**Mean**	**Lower**	**Upper**	**Distribution**	**Source**
**Cost of drugs**					
Camrelizumab/cycle	460.31	230.15	460.31	gamma	(29, 36)
Carboplatin/cycle	40.67	40.60	43.30	gamma	
Paclitaxel/cycle	105.09	105.03	105.09	gamma	
Docetaxel/cycle	32.57	31.69	33.82	gamma	
Best supportive care/cycle	338.00	159.00	476.00	gamma	(19)
**Cost of hospitalization**					
Cost of CT examination/1 time	58.17	45.99	68.98	gamma	(29)
Cost of blood biochemical examination/1 time	47.05	37.20	55.80	gamma	
Cost of blood test/1 time	3.14	2.49	3.73	gamma	
Cost of urinalysis/1 time	0.63	0.50	0.75	gamma	
Cost of diagnosis	3.14	1.55	4.66	gamma	
Cost of intravenous injection	1.73	1.55	2.14	gamma	
Cost of care	3.77	2.98	4.47	gamma	
Cost of bed	6.60	5.22	7.83	gamma	
Cost of end-of-life	2325.75	1860.60	2790.90	gamma	(28)
**Cost of AE**					
Cost of neutrophil count decreased	116.37	51.11	357.80	gamma	(28)
Cost of white blood cell count decreased	116.37	51.11	357.80	gamma	
Cost of platelet count decreased	1523.82	1240.17	1771.67	gamma	
Cost of anemia	140.40	106.73	160.10	gamma	
Cost of pneumonia	6491.17	5192.94	7789.40	gamma	(37)
**Utility**					
Utility of progression-free survival	0.86	0.83	0.88	beta	(31)
Utility of disease progression	0.32	0.26	0.39	beta	(38)
**Disutility of AE**					
Disutility of neutrophil count decreased	0.20	0.16	0.24	beta	(31)
Disutility of white blood cell count decreased	0.20	0.16	0.24	beta	
Disutility of platelet count decreased	0.11	0.09	0.13	beta	(32)
Disutility of anemia	0.07	0.06	0.09	beta	(39)
Disutility of pneumonia	0.05	0.04	0.06	beta	(40)
**Risk of AE**					
**Camrelizumab plus chemotherapy group**					
neutrophil count decreased	0.07	0.05	0.08	beta	(15)
white blood cell count decreased	0.30	0.24	0.36	beta	
platelet count decreased	0.55	0.44	0.67	beta	
anemia	0.10	0.08	0.12	beta	
**Chemotherapy group**					
neutrophil count decreased	0.26	0.21	0.31	beta	
white blood cell count decreased	0.59	0.47	0.71	beta	
pneumonia	0.05	0.04	0.06	beta	
anemia	0.07	0.06	0.09	beta	
**Time duration of AE**					
Time duration of neutrophil count decreased	4.19	3.35	5.03	normal	Expert consultation
Time duration of anemia	6.83	5.46	8.20	normal	
Time duration of white blood cell count decreased	4.50	3.60	5.40	normal	
Time duration of platelet count decreased	47.29	37.83	56.75	normal	
Time duration of pneumonia	21.00	16.80	25.20	normal	
**Proportions of subsequent treatment**					
Subsequent chemotherapy proportions of camrelizumab plus chemotherapy group	0.34	0.27	0.40	beta	(14, 15)
Crossover proportions of chemotherapy group	0.47	0.37	0.56	beta	
Subsequent chemotherapy proportions of chemotherapy group	0.11	0.09	0.14	beta	
Discount rate	0.05	0.00	0.08	beta	(23)
**Clinical input**					
**Survival models for camrelizumab plus chemotherapy group**					
Fractional polynomial for OS	Power =–1, alpha = −0.16843; intercept = −0.7434				
Royston-Parmar spline models for PFS	Scale = normal, gamma0 = 0.2758, gamma1 = 0.9662				
**Survival models for chemotherapy group**					
Fractional polynomial for OS	Power = −1, alpha = −0.31955; intercept = 0.10144				
Royston-Parmar spline models for PFS	Scale = normal, gamma0 = 1.962, gamma1 = 1.638,				
	gamma2 = 3.834, gamma3 =-10.048, gamma4 = 7.198				

Costs are in USD; AE, adverse events; OS, overall survival; PFS, progression free survival.

### Cost

From the perspective of the Chinese healthcare system, only direct medical costs were considered, including costs of acquiring drugs, costs attributed to the patient's diagnosis and hospitalization, costs for the management of adverse events (AEs), and costs for end-of-life care (Eol) were analyzed (28). Drug prices were obtained from public databases and were all up to date in 2021 (29, 30). Since carboplatin and paclitaxel had multiple dosage forms in Chinese market, we chose the most reasonable dosage combination which meet the balance of both effect and lower cost. For example, a patient needed 344 mg paclitaxel per cycle, with two dosage forms available: 30 mg and 100 mg (unit price of 2 dosage forms are equal). A reasonable dosage combination would be 2 30 mg plus 3 100 mg. Thus, the cost of first-line combination therapy per cycle (21 days) would be $606.07 ($460.31 per cycle for camrelizumab, $105.09 per cycle for 344 mg paclitaxel, and $40.67 per cycle for 475 mg carboplatin). We only considered severe AEs (≥grade 3) with rates over 5%, including white blood cell count decreased, neutrophil count decreased, anemia, pneumonia, and platelet count decreased (15). It cannot be ignored that some immune-related AEs such as reactive cutaneous capillary endothelial proliferation (RCCEP) and hypothyroidism were also common. We did not include them because their occurence of grades 3–5 were relative low with 2 and 1%, respectively (15). Costs of AEs were extracted from published articles and duration of AEs were available from expert consultation (28). All the cost-related parameters are shown in Table 1.

### Utility

The PFS and PD states associated with advanced sq-NSCLC were 0.86 and 0.32, respectively, which were derived from two health state utilities researches on China patients with NSCLC. The disutility values because of the AEs were included in this analysis and were extracted from other studies (31, 32, 39). All the AEs were assumed to be incurred during the first cycle (21). The duration-adjusted disutility was subtracted from the baseline PFS utility. All the utility-related parameters are shown in Table 1.

### Sensitivity analysis

Sensitivity analysis were conducted to test the robustness of the model. In deterministic sensitivity analysis (DSA), all the parameters were adjusted within the reported 95% CIs or assuming reasonable ranges of the base–case values (±20%). A Monte Carlo simulation was performed for 10,000 iterations and we conducted probabilistic sensitivity analysis (PSA). A gamma distribution was selected for cost and a beta distribution for probability, proportion, and utility (21). We used scatter plot and cost-effectiveness acceptability curves (CEACs) to analyze the cost-effectiveness for each regimen with various willingness to pay (WTP) threshold.

For scenario analysis 1, since OS data in CameL-sq was not mature, its OS data of chemotherapy from 18th month to the termination was bridged by the OS data of chemotherapy of KEYNOTE-407 (8). Then, the OS of combination therapy from the 18th month to the termination of the model was estimated to verify the base–case analysis results (21). For scenario analysis 2, we considered that the price of camrelizumab fluctuated between 0.5 times and 2 times of its price in NRDL, since camrelizumab treating advanced sq-NSCLC had not been listed in NRDL. For scenario analysis 3, taking the uncertainty of subsequent treatment into account, in addition to the docetaxel, patients may also choose other drugs, such as immunotherapy and targeted therapy. So, we assumed subsequent treatment unit cost range from $30~$1,500 to test the robustness of base–case analysis results.

In the subgroup analysis, the ICER was calculated for each subgroup using the subgroup specific HRs for OS and PFS obtained from CameL-sq. We considered the subgroup of patients with different ages, Eastern Cooperative Oncology Group (ECOG) performance status score, disease stage, and PD-L1 tumor proportion score. Data for all the subgroups except for the HRs for OS and PFS were assumed to be the same since the lack of sufficient data, and proportional hazards was assumed.

## Results

### Base–case analysis results

Results of base–case analysis are shown in Table 2. The cumulative cost of camrelizumab plus chemotherapy were significantly higher than chemotherapy for both OS and PFS ($19,165.08 vs. $12,817.27 and $8,768.36 vs. $2,206.55). Camrelizumab plus chemotherapy was associated with an improvement of 0.91 life-years (2.38 vs. 1.47 life-years) and 0.47 QALYs (1.12 vs. 0.65 QALYs). The ICER for camrelizumab plus chemotherapy compared with chemotherapy was $13,572 per QALY, which was slightly higher than 1 time GDP per capita.

**Table 2:** Results of base–case analysis and scenario analysis.

**Drug**	**Total**			**Only PFS**			**Total**		
	**Cost**	**Life-years**	**Utility**	**Cost**	**Life-years**	**Utility**	**Increment cost**	**Increment utility**	**ICER**
base–case analysis									
Chemotherapy	12817.27	1.47	0.65	2206.55	0.36	0.30	——	——	——
Camrelizumab plus chemotherapy	19165.08	2.38	1.12	8768.36	0.78	0.65	6347.81	0.47	13571.68
scenario analysis 1									
Chemotherapy	14026.06	1.65	0.70	2206.55	0.36	0.30	——	——	——
Camrelizumab plus chemotherapy	20119.08	2.59	1.17	8768.36	0.78	0.65	6093.02	0.47	12886.09

PFS, progression free survival; ICER, incremental cost-effectiveness ratio.

### Sensitivity analysis

**Figure 1:**
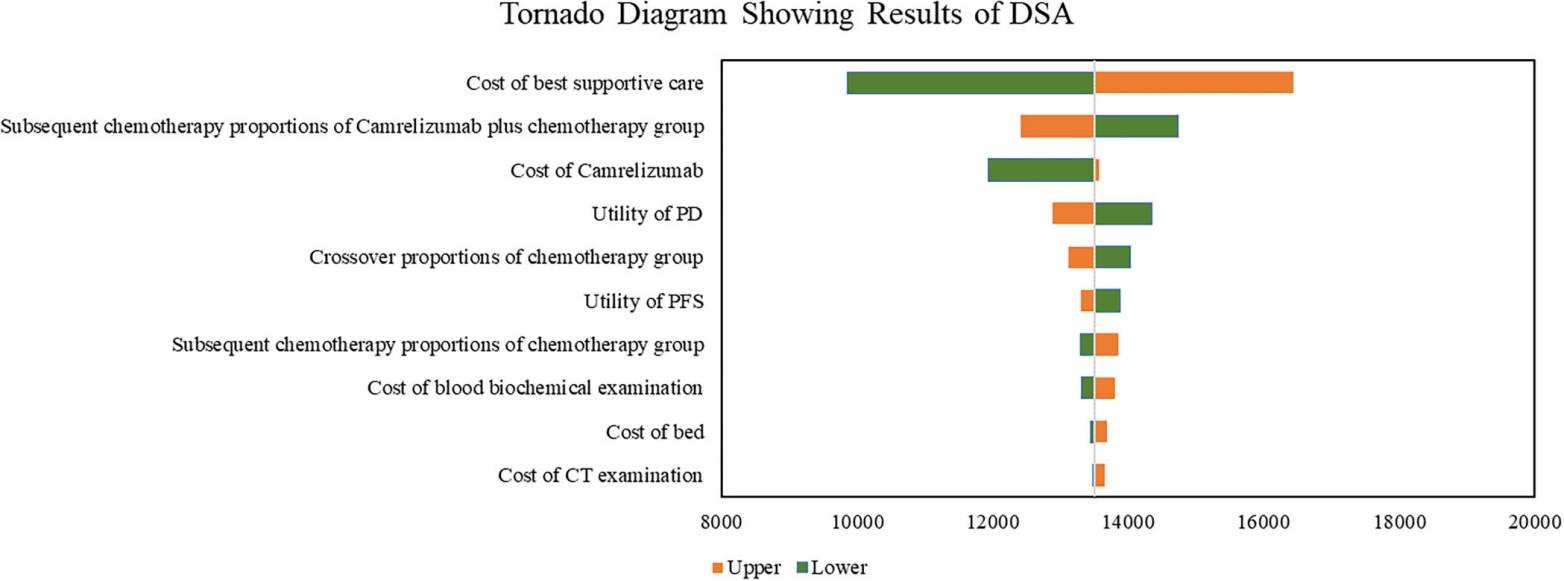
Tornado diagram showing results of DSA. DSA, deterministic sensitivity; PD, progression disease; PFS, progression-free survival.

Results of DSA are shown in Figure 1. Cost of best supportive care was the factor with the greatest influence, followed by subsequent chemotherapy proportions of camrelizumab plus chemotherapy group, cost of camrelizumab, utility of PD, and the crossover proportions of chemotherapy. With all the parameters fluctuating in the upper and lower limits, the results were consistent with the base–case analysis, indicating that our base–case analysis results were relatively stable as a whole.

**Figure 2:**
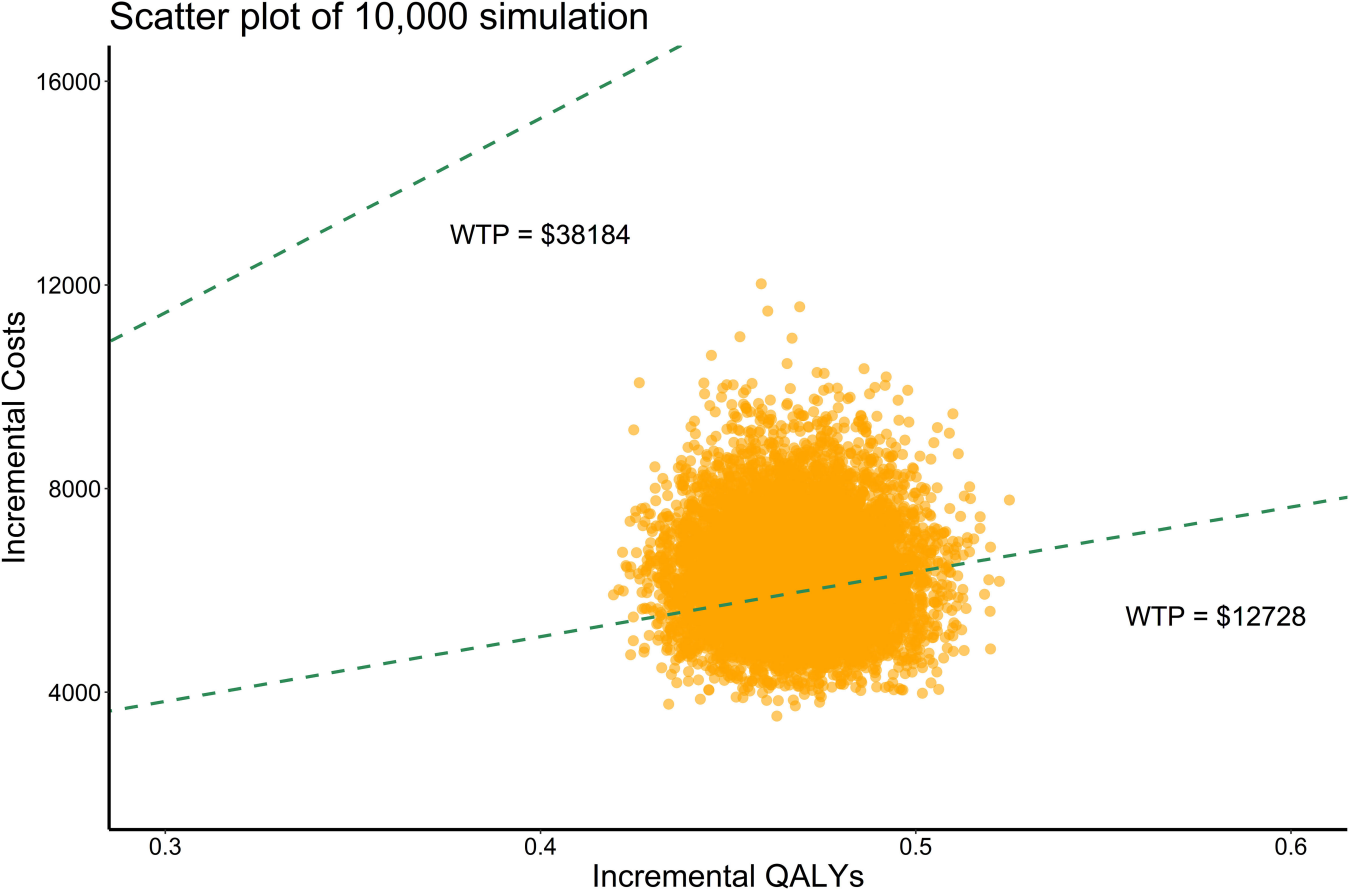
Probabilistic sensitivity analysis, scatter plot (10,000 iterations). WTP, willingness-to-pay; QALYs, quality-adjusted life years.

**Figure 3:**
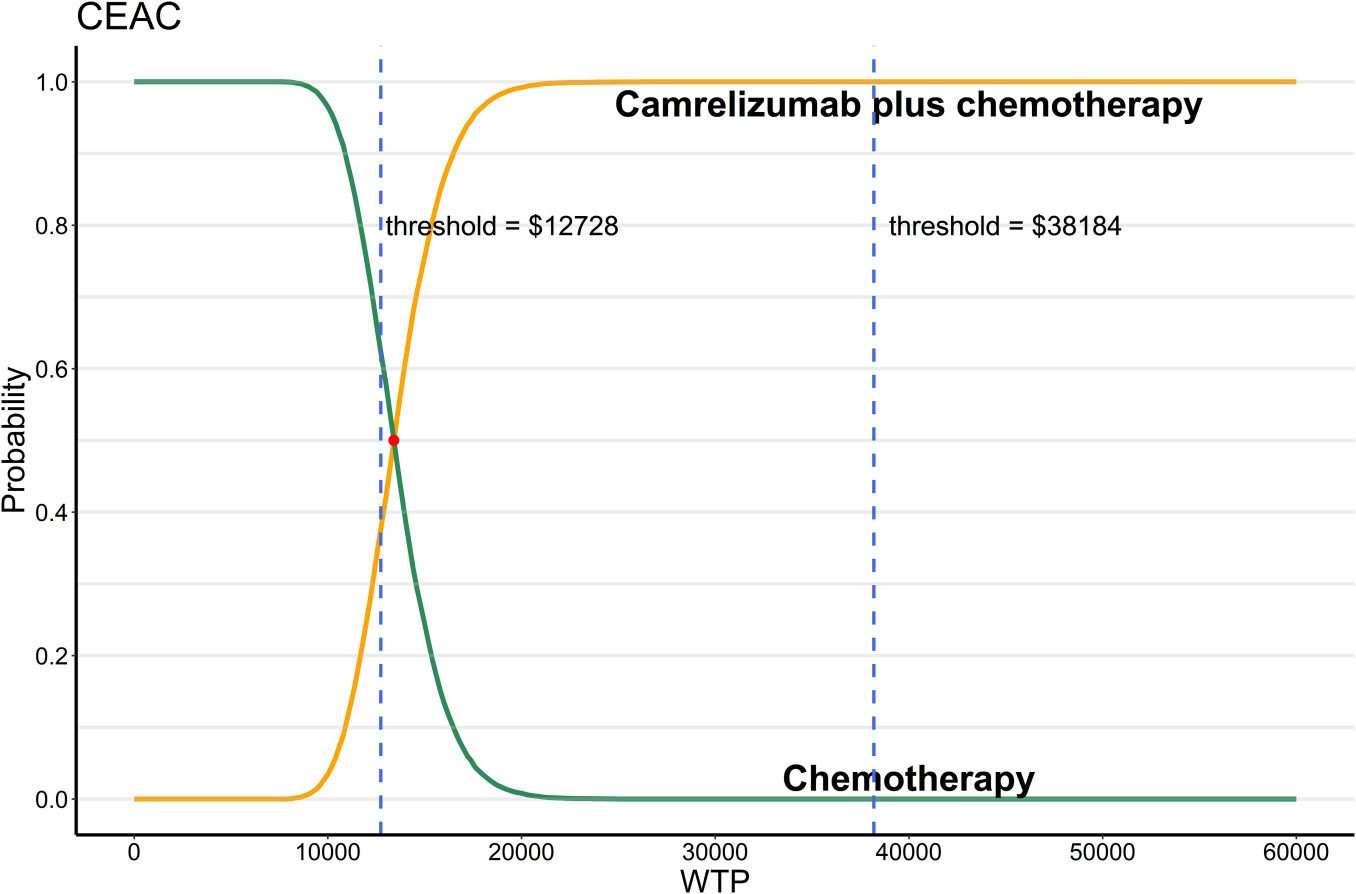
Probabilistic sensitivity analysis, cost-effectiveness acceptability curve (10,000 iterations). WTP, willingness-to-pay; CEAC, cost-effectiveness acceptability curve.

As shown in Figure 2, when WTP equals to 1 time GDP per capita, almost two thirds of the scatter points were above the line of WTP. When WTP equals to 3 times GDP per capita, all scatter points of ICER were below the line of WTP. The CEAC (Figure 3) showed that when WTP ranged from $12,728 to $38,184 (1 times to 3 times GDP per capita) per QALY, the probability of camrelizumab plus chemotherapy being cost-effective increased from 37.8 to 100%.

### Scenario analysis

**Figure 4:**
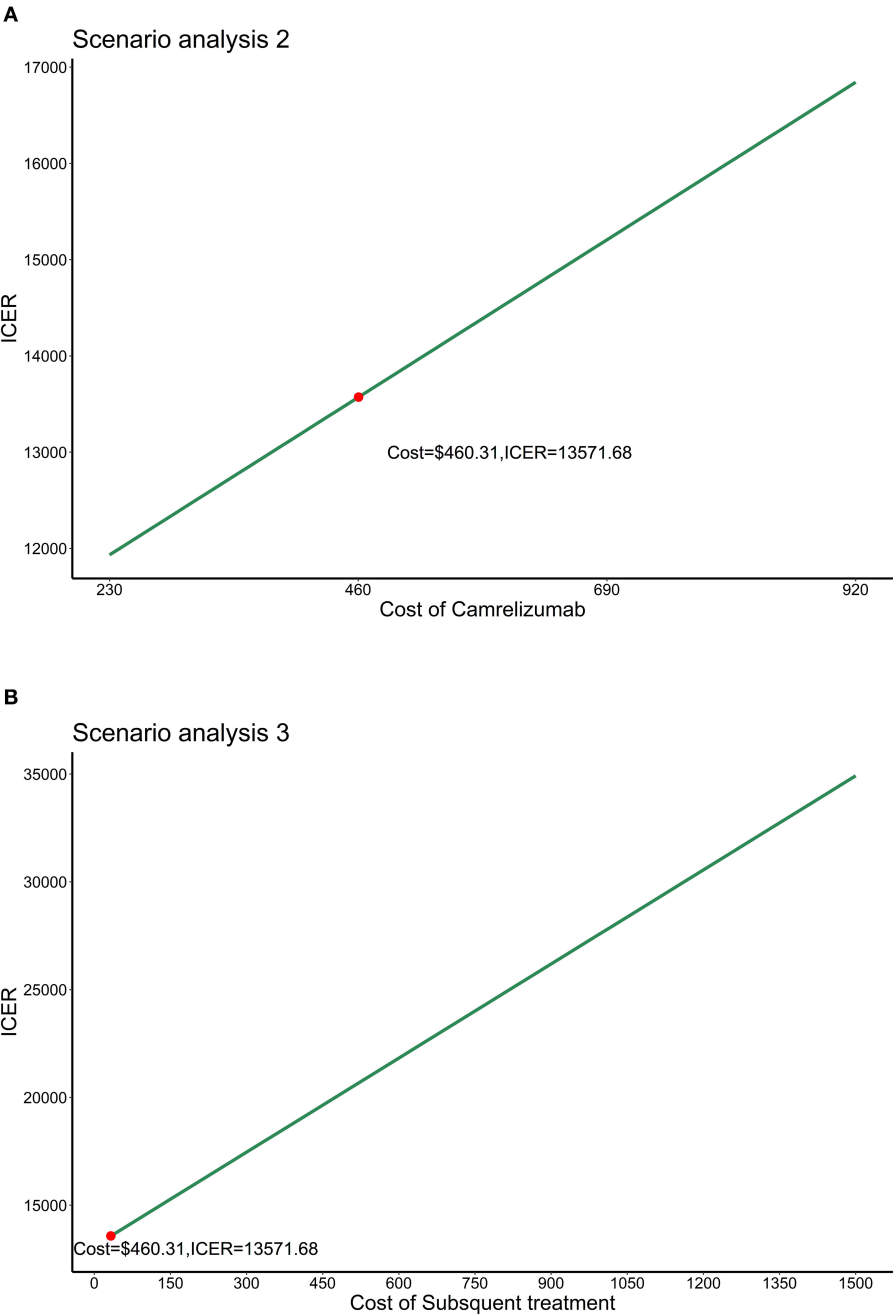
Results of scenario analysis 2 and 3. **(A)** Results of scenario analysis 2. **(B)** Results of scenario analysis 3. ICER, Incremental cost-effectiveness ratio.

In scenario analysis 1, the results showed that cost, QALYS and life-years gained were close to the base–case analysis results. The ICER was estimated to be $12,886 per QALY. In scenario analysis 2, we allowed the price of camrelizumab fluctuated in the range of $230~$920 (0.5 times to 2 times the current price) with other parameters unchanged. ICER would increase with the increase in the price of camrelizumab. ICERs were all below the WTP threshold of 3 times GDP per capita as presented in Figure 4A. Allowed subsequent treatment with drugs other than docetaxel in scenario analysis 3, Figure 4B showed that when the unit cost of subsequent treatment ranged from $30 to $1,500, ICERs were all below the WTP threshold of 3 times GDP per capita.

### Subgroup analysis

**Figure 5:**
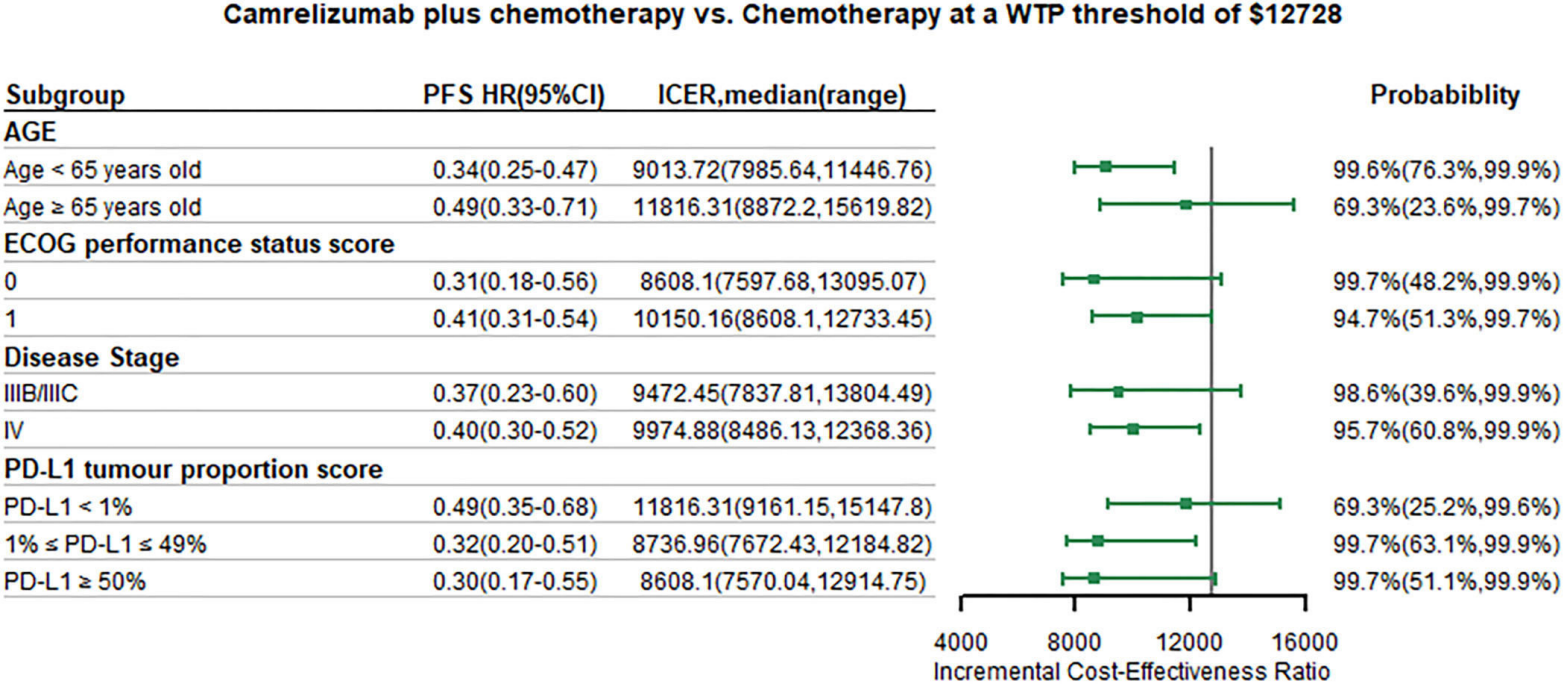
Subgroup analysis results of incremental cost-effectiveness ratio (ICER) and probabilities of cost-effectiveness obtained by varying the hazard ratios (HRs) for progression-free survival. WTP, willingness-to-pay; PFS, progression-free survival; HR, hazard ratio; CI, confidence interval; ICER, Incremental cost-effectiveness ratio.

**Figure 6:**
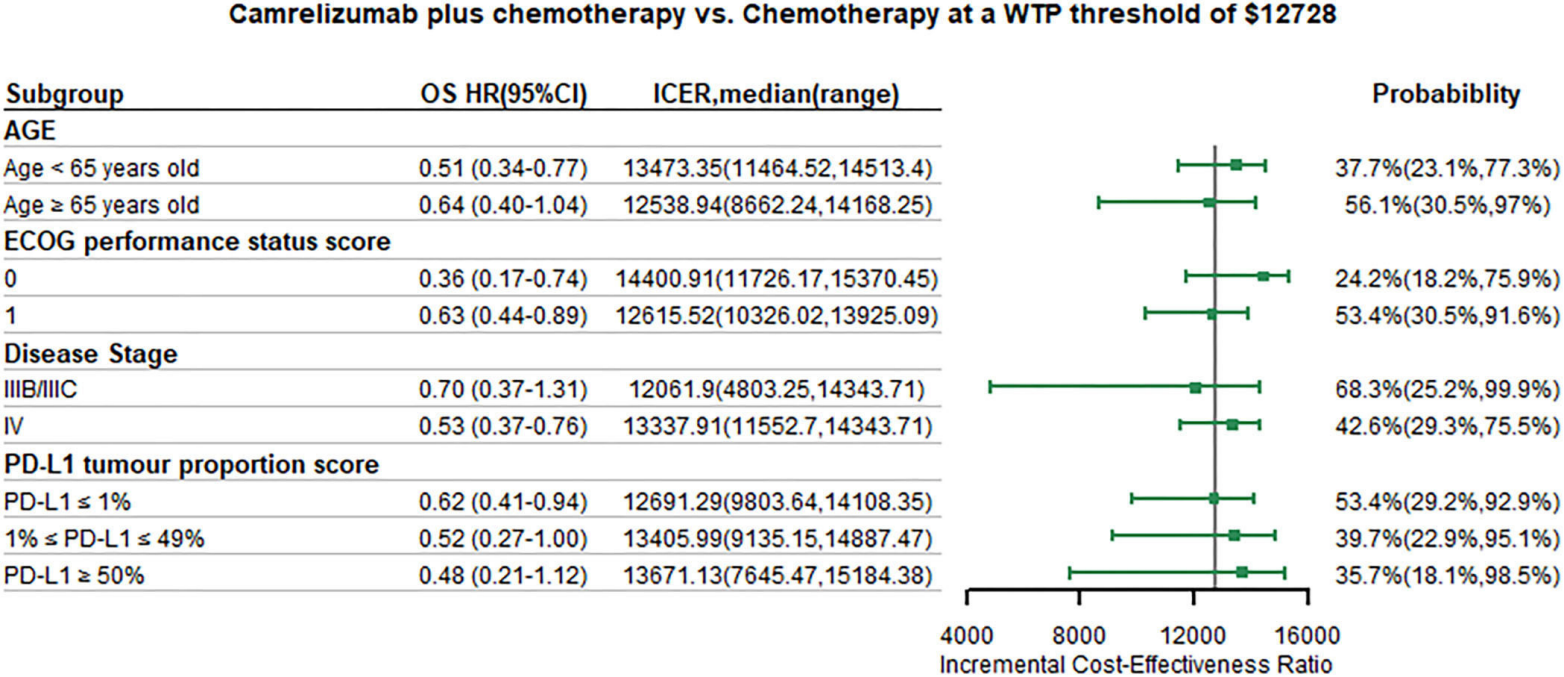
Subgroup analysis results of incremental cost-effectiveness ratio (ICER) and probabilities of cost-effectiveness obtained by varying the hazard ratios (HRs) for overall survival. WTP, willingness-to-pay; OS, overall survival; HR, hazard ratio; CI, confidence interval; ICER, Incremental cost-effectiveness ratio.

We conducted subgroup analysis by varying the HR of PFS and OS at the WTP threshold of 1 time GDP per capita. Summary results of subgroup analysis are concluded in Figures 5, 6. The results of subgroup analysis by varying the HR of OS showed that the following subgroups were associated with positive ICER and > 50% probability to be cost-effective: patients older than 65 years old, patients with ECOG performance status score equals to 1, patients with disease stage IIIB/IIIC, and patients with PD-L1 tumor proportion score ≤ 1%. The results of subgroup analysis by varying the HR of PFS showed that all subgroups were associated with positive ICER and the probability to be cost-effective were > 50%.

## Discussion

CameL-sq is the first study to sheds some light on the potential impact on survival of immunotherapy in the Chinese population with sq-NSCLC (33), with previous studies did not provide enough evidence. Our study addresses the unmet need for an economic evaluation of camrelizumab plus chemotherapy. Our analysis showed that camrelizumab plus chemotherapy was unfavorable with a WTP thresholds lower than $13,410. When WTP thresholds raised to 3 times GDP per capita, combination therapy was significant cost-effective. This finding was robust, as shown by the results of DSA and PSA. At a WTP threshold of $12,728 per QALY, 4 subgroups were associated with positive ICER and > 50% probability of being cost-effective, namely, patients older than 65 years old, ECOG performance status score equals to 1, disease stage IIIB/IIIC and PD-L1 tumor proportion score ≤ 1%. Scenario analysis showed that by using bridged data of chemotherapy of KEYNOTE-407, our results of base–case analysis was robust. By increasing the cost of camrelizumab and subsequent treatment, ICER would increase too.

According to our base–case analysis results, camrelizumab plus chemotherapy was not favorable compared with chemotherapy with a WTP threshold of $12,728 per QALY. However, when WTP threshold raised to $38,184 per QALY, combination therapy had a probability close to 100% to be cost-effectiveness. This meant that for patients with sq-NSCLC with a higher willingness-to-pay, camrelizumab plus chemotherapy would be a potentially effective and cost-effective option. But for patients with lower willingness-to-pay, camrelizumab still needs to lower the price to achieve cost-effectiveness. The current price of camrelizumab is $460.31 per cycle according to the latest Chinese healthcare negotiation which was considered in this study (29, 30). However, the first-line therapy of camrelizumab plus chemotherapy in sq-NSCLC was not listed in the NRDL. This meant that the affordability of this new treatment for patients were still unknown. Currently, the price of camrelizumab is $3,113.2 per cycle for indications that are not covered by health insurance. The Newest Patient Assistance Program for camrelizumab is “2+2 then 4+n (No more than 1 year)” (34). When camrelizumab was not listed in NRDL, patients' affordability for camrelizumab would be greatly reduced, and both the manufacturer and the government did not want this to happen. But, we considered that if camrelizumab was listed in NRDL, the price would be at most $460.31 per cycle, which meant that the baseline cost of camrelizumab considered in this study was reasonable. Since the price of camrelizumab was still not sure with great uncertainty, we considered a wide range of the price of camrelizumab to roughly evaluate the patient's accessibility to the treatment. Results showed that ICER was positively correlated with the price of camrelizumab. These findings would provide evidence to help Chinese policy makers to judge whether camrelizumab was suitable to be listed as a first-line therapy for sq-NSCLC in NRDL. However, the calculation process in this study can be far from accurate since we did not consider any reimbursement policies. Therefore, future budget impact analysis are still needed to evaluate the patient's affordability to the new treatment.

Results of subgroup analysis indicated that camrelizumab plus chemotherapy was more cost-effective for patients with specific baseline characteristics, such as patients older than 65 years old, patients with ECOG performance status score equals to 1, patients with disease stage IIIB/IIIC and patients with PD-L1 tumor proportion score ≤ 1%. These findings can help clinicians tailoring treatments based on individual patient factors. The DSA results showed that cost of best supportive care, subsequent chemotherapy proportions of camrelizumab plus chemotherapy group and cost of camrelizumab were the most influential parameters. With the wide range of these parameters, combination therapy would still have significant cost-effectiveness with a WTP threshold of 3 times GDP per capita. Since the subsequent treatment of patients in CameL-sq was still unknown, we assumed patients only receive chemotherapy, best supportive care or crossover to combination therapy (only for chemotherapy group). The proportion was estimated according to CameL trial (14, 19). Cost of the best supportive care and subsequent chemotherapy proportions do had large influence since these two parameters would greatly affect the total cost of disease progression.

To our knowledge, this study is the first to evaluate the economic outcomes of camrelizumab plus carboplatin and paclitaxel as first-line treatment for advanced sq-NSCLC by synthesizing the latest evidence through an economic modeling approach. Sq-NSCLC is still a clinical trouble in China. Camrelizumab may opening a window of opportunity for patients with Sq-NSCLC to achieve overall survival benefit. This study provides evidence of cost-effectiveness which may accelerate the process of listing in health insurance and promotion. Second, findings of this study were robust according to our sensitivity analysis and scenario analysis. We considered flexible parametric models to fit and extrapolate the survival data which was more accurate than standard survival models. Economic information for the subgroups may help treatment decisions making for physicians, patients, and policy makers.

There are several limitations in the study. First, due to the lack of head-to-head data, we did not include other Chinese immune checkpoint inhibitors, namely, tislelizumab and sintilimab, which have shown significant PFS benefits but with OS benefits still unknown. Second, the long-term benefits of camrelizumab plus carboplatin and paclitaxel for sq-NSCLC remains a question. With many information still known for this 2-year follow-up, long-term efficacy, and subsequent treatment were all the estimated in this model. This may bring uncertainty although the model and parameters were validated. Third, the utilities in the model were not estimated from CameL-sq, but from other health utility surveys in patients with NSCLC. In addition, we assumed the same utility for patients in both groups, which may bring some bias to the results of cost-effectiveness analysis. Forth, we did not consider the immune-related AEs and grades 1 or 2 AEs, which may overestimated the results associated with camrelizumab plus carboplatin and paclitaxel. This limitation may not have a major influence, as suggested by the findings in the DSA indicating that the costs and disutilities associated with AEs were minor. However, these AEs cannot be ignored in general clinical practice. Fifth, the cost of pneumonia management and the end-of-life cost were extracted from studies conducted in developed countries, which might lead to bias when directly applied in the setting of China in this study. Therefore, for pneumonia management cost, we considered a potential alternative value from a study targeted on stage III non-small cell lung cancer in China to test the uncertainty (35). The baseline value of the cost of pneumonia management was changed from $6491.17 per cycle to $1,640 per cycle, then the ICER changed from $13,572 per QALY to $12988.96 per QALY. Thus, this limitation did not lead to a significant change to the ICER which was still over the given WTP. For the end-of-life cost, although there were no alternative value, according to the DSA, the end-of-life cost have little influence on ICER. However, the cost of pneumonia management and end-of-life cost in treating Chinese advanced patients with NSCLC with immunotherapy still needs further study.

## Conclusion

The findings of this economic evaluation suggest that from the perspective of the Chinese healthcare system, camrelizumab plus carboplatin and paclitaxel would unlikely to be a cost-effective option at a WTP threshold of 1 time GDP per capita. But the economic outcomes can be improved in patients with specific baseline characteristics. These results may help clinicians in making optimal decisions in treating advanced sq-NSCLC. However, because of the several limitations in this study, further long-term follow-up data and real-world data are needed.

## Data availability statement

The original contributions presented in the study are included in the article/Supplementary material, further inquiries can be directed to the corresponding author.

## Author contributions

Conceptualization: TS and WT. Methodology: TS, YR, and MZ. Analysis, visualization, and writing—original draft preparation: TS and MZ. Writing–review and editing: YR and WT. Funding acquisition: WT. Supervision: MZ and WT. All the authors read and approved the final manuscript.

## Funding

The General Program of National Natural Science Foundation of China (72174207).

## Conflict of interest

The authors declare that the research was conducted in the absence of any commercial or financial relationships that could be construed as a potential conflict of interest.

## Publisher's note

All claims expressed in this article are solely those of the authors and do not necessarily represent those of their affiliated organizations, or those of the publisher, the editors and the reviewers. Any product that may be evaluated in this article, or claim that may be made by its manufacturer, is not guaranteed or endorsed by the publisher.
