# Supplementary material for: Cost-effectiveness analysis of camrelizumab plus chemotherapy as first-line treatment for advanced squamous NSCLC in China
Source: Front Public Health. 2022 Aug 15;10:912921. doi: 10.3389/fpubh.2022.912921 (PMC9423383; doi:10.3389/fpubh.2022.912921)
Supplement: Supplementary file 1 [file Data_Sheet_1.docx]

***Supplementary Materials***

Content

[1. Goodness-of-fit of different models (Tables for Statistical results and Figures for survival curves) 2](#_Toc12461)

[1.1 OS of Camrelizumab 2](#_Toc2360)

[1.2 OS of chemotherapy 4](#_Toc2577)

[1.3 PFS of Camrelizumab 6](#_Toc12431)

[1.4 PFS of chemotherapy 8](#_Toc10405)

[1.5 OS of Pembrolizumab 10](#_Toc2462)

[1.6 Explanation to the above figures and tables 12](#_Toc10040)

[1.7 Explanation of methodology used in fit and extrapolate survival outcomes 12](#_Toc30798)

[2. The Replicated Kaplan-Meier PFS Curves 14](#_Toc1297)

[3. The Replicated Kaplan-Meier OS Curves 15](#_Toc9640)

[4. CHEERS 2022 Checklist 16](#_Toc23151)

## Goodness-of-fit of different models (Tables for Statistical results and Figures for survival curves)

### 1.1 OS of Camrelizumab

| No | model | Knots/power | LnL | Params | AIC | Rank |
| --- | --- | --- | --- | --- | --- | --- |
| 1 | exp | / | -41.01 | 1 | 84.03 | 20 |
| 2 | weibull | / | -35.52 | 2 | 75.03 | 11 |
| 3 | gamma | / | -35.14 | 2 | 74.27 | 9 |
| 4 | lnorm | / | -34.51 | 2 | 73.01 | 4 |
| 5 | gompertz | / | -37.61 | 2 | 79.23 | 18 |
| 6 | llogistic | / | -35.09 | 2 | 74.18 | 7 |
| 7 | gengamma | / | -34.52 | 3 | 75.03 | 13 |
| 8 | genf | / | -34.52 | 4 | 77.03 | 16 |
| ***9*** | ***FP1_1*** | ***-1*** | ***-34.06*** | ***2*** | ***72.12*** | ***1*** |
| 10 | FP1_2 | -2 | -34.12 | 2 | 72.24 | 2 |
| 11 | FP2_1 | -2;0.5 | -33.79 | 3 | 73.58 | 5 |
| 12 | FP2_2 | -2;1 | -33.80 | 3 | 73.59 | 6 |
| 13 | RCS1 | 1 | -36.22 | 3 | 78.43 | 17 |
| 14 | RCS2 | 2 | -36.11 | 4 | 80.21 | 19 |
| 15 | RP_hazard_1 | 0 | -35.52 | 2 | 75.03 | 12 |
| 16 | RP_hazard_2 | 1 | -34.85 | 3 | 75.70 | 14 |
| 17 | RP_odds_1 | 0 | -35.09 | 2 | 74.18 | 8 |
| 18 | RP_odds_2 | 1 | -34.88 | 3 | 75.75 | 15 |
| 19 | RP_lnorm_1 | 0 | -34.51 | 2 | 73.01 | 3 |
| 20 | RP_lnorm_2 | 1 | -34.48 | 3 | 74.95 | 10 |


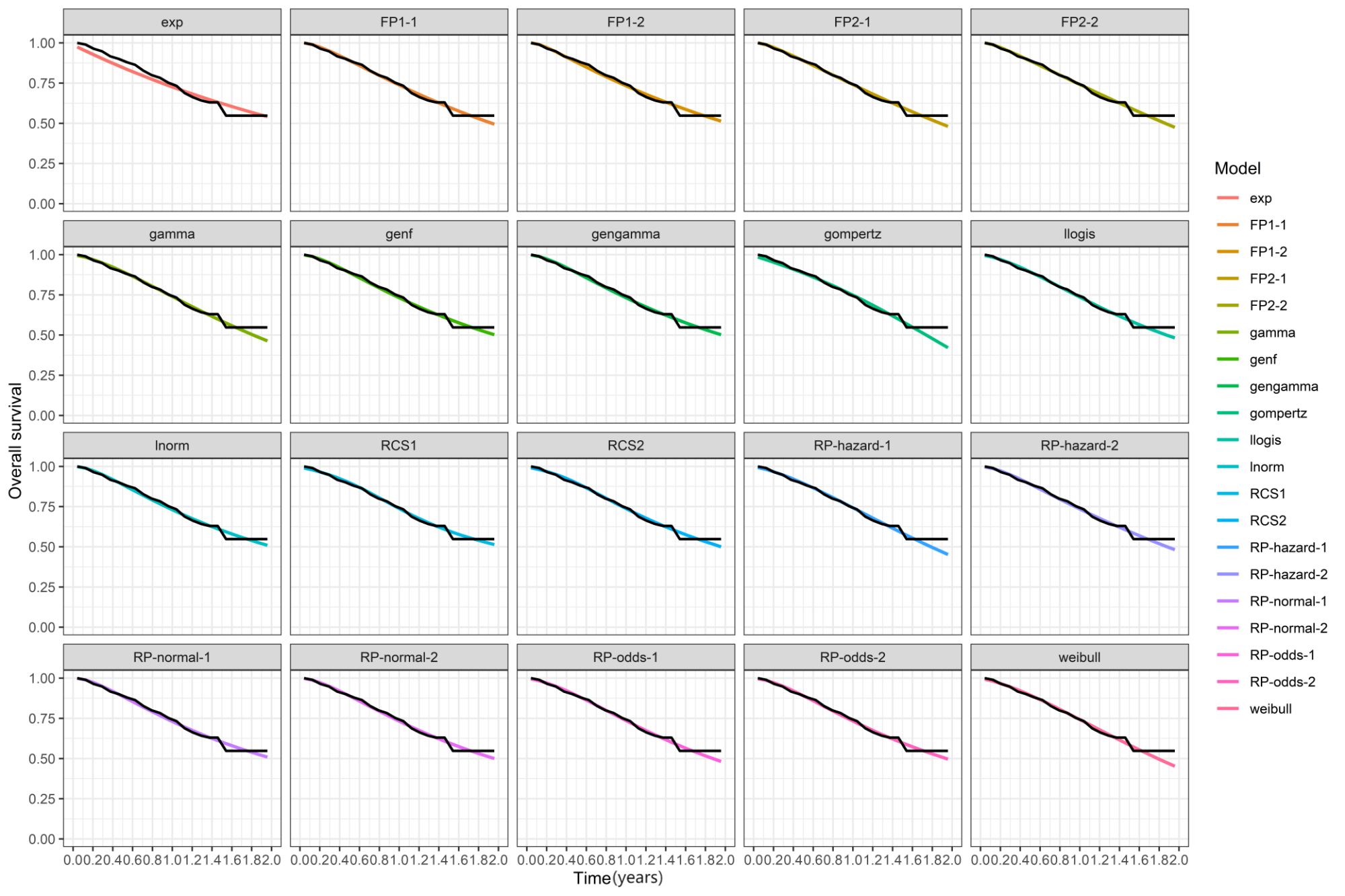


### 1.2 OS of chemotherapy

| No | model | Knots/power | LnL | Params | AIC | Rank |
| --- | --- | --- | --- | --- | --- | --- |
| 1 | exp | / | -56.76 | 1 | 115.53 | 20 |
| 2 | weibull | / | -40.24 | 2 | 84.47 | 16 |
| 3 | gamma | / | -38.75 | 2 | 81.50 | 9 |
| 4 | lnorm | / | -37.76 | 2 | 79.52 | 3 |
| 5 | gompertz | / | -46.24 | 2 | 96.49 | 19 |
| 6 | llogistic | / | -38.55 | 2 | 81.10 | 8 |
| 7 | gengamma | / | -37.82 | 3 | 81.64 | 11 |
| 8 | genf | / | -37.84 | 4 | 83.67 | 14 |
| ***9*** | ***FP1_1*** | ***-1*** | ***-37.49*** | ***2*** | ***78.98*** | ***1*** |
| 10 | FP1_2 | -0.5 | -38.03 | 2 | 80.06 | 4 |
| 11 | FP2_1 | -0.5;-0.5 | -37.47 | 3 | 80.94 | 5 |
| 12 | FP2_2 | -0.5;0 | -37.47 | 3 | 80.95 | 6 |
| 13 | RCS1 | 1 | -41.38 | 3 | 88.75 | 18 |
| 14 | RCS2 | 2 | -39.88 | 4 | 87.76 | 17 |
| 15 | RP_hazard_1 | 1 | -37.94 | 3 | 81.88 | 12 |
| 16 | RP_hazard_2 | 0 | -40.24 | 2 | 84.47 | 15 |
| 17 | RP_odds_1 | 0 | -38.55 | 2 | 81.10 | 7 |
| 18 | RP_odds_2 | 1 | -38.07 | 3 | 82.14 | 13 |
| 19 | RP_lnorm_1 | 0 | -37.76 | 2 | 79.52 | 2 |
| 20 | RP_lnorm_2 | 1 | -37.81 | 3 | 81.62 | 10 |


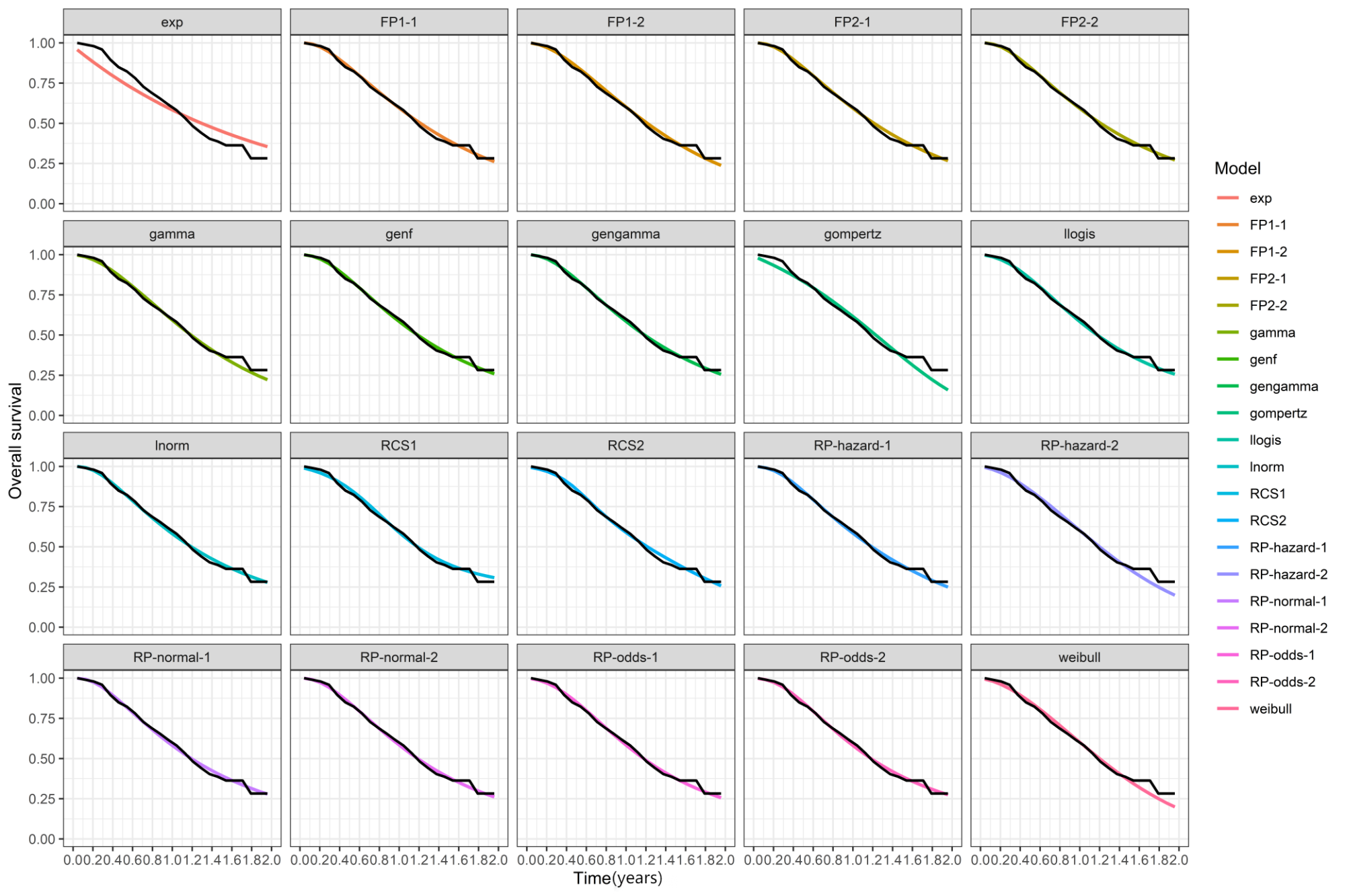


### 1.3 PFS of Camrelizumab

| No | model | Knots/power | LnL | Params | AIC | Rank |
| --- | --- | --- | --- | --- | --- | --- |
| 1 | exp | / | -63.15 | 1 | 128.29 | 19 |
| 2 | weibull | / | -59.35 | 2 | 122.70 | 17 |
| 3 | gamma | / | -57.73 | 2 | 119.47 | 16 |
| ***4*** | ***lnorm*** | ***/*** | ***-52.32*** | ***2*** | ***108.64*** | ***2*** |
| 5 | gompertz | / | -62.66 | 2 | 129.32 | 20 |
| 6 | llogistic | / | -54.25 | 2 | 112.50 | 12 |
| 7 | gengamma | / | -52.20 | 3 | 110.41 | 6 |
| 8 | genf | / | -52.37 | 4 | 112.73 | 13 |
| 9 | FP1_1 | -2 | -53.49 | 2 | 110.98 | 9 |
| 10 | FP1_2 | -1 | -55.30 | 2 | 114.60 | 14 |
| 11 | FP2_1 | -1;1 | -52.45 | 3 | 110.89 | 7 |
| 12 | FP2_2 | -1;0.5 | -52.47 | 3 | 110.94 | 8 |
| 13 | RCS1 | 1 | -58.78 | 3 | 123.56 | 18 |
| 14 | RCS2 | 2 | -53.71 | 4 | 115.42 | 15 |
| 15 | RP_hazard_1 | 1 | -51.98 | 3 | 109.96 | 3 |
| 16 | RP_hazard_2 | 2 | -51.79 | 4 | 111.59 | 10 |
| 17 | RP_odds_1 | 1 | -52.16 | 3 | 110.32 | 4 |
| 18 | RP_odds_2 | 0 | -54.25 | 2 | 112.50 | 11 |
| ***19*** | ***RP_lnorm_1*** | ***0*** | ***-52.32*** | ***2*** | ***108.64*** | ***1*** |
| 20 | RP_lnorm_2 | 1 | -52.18 | 3 | 110.36 | 5 |


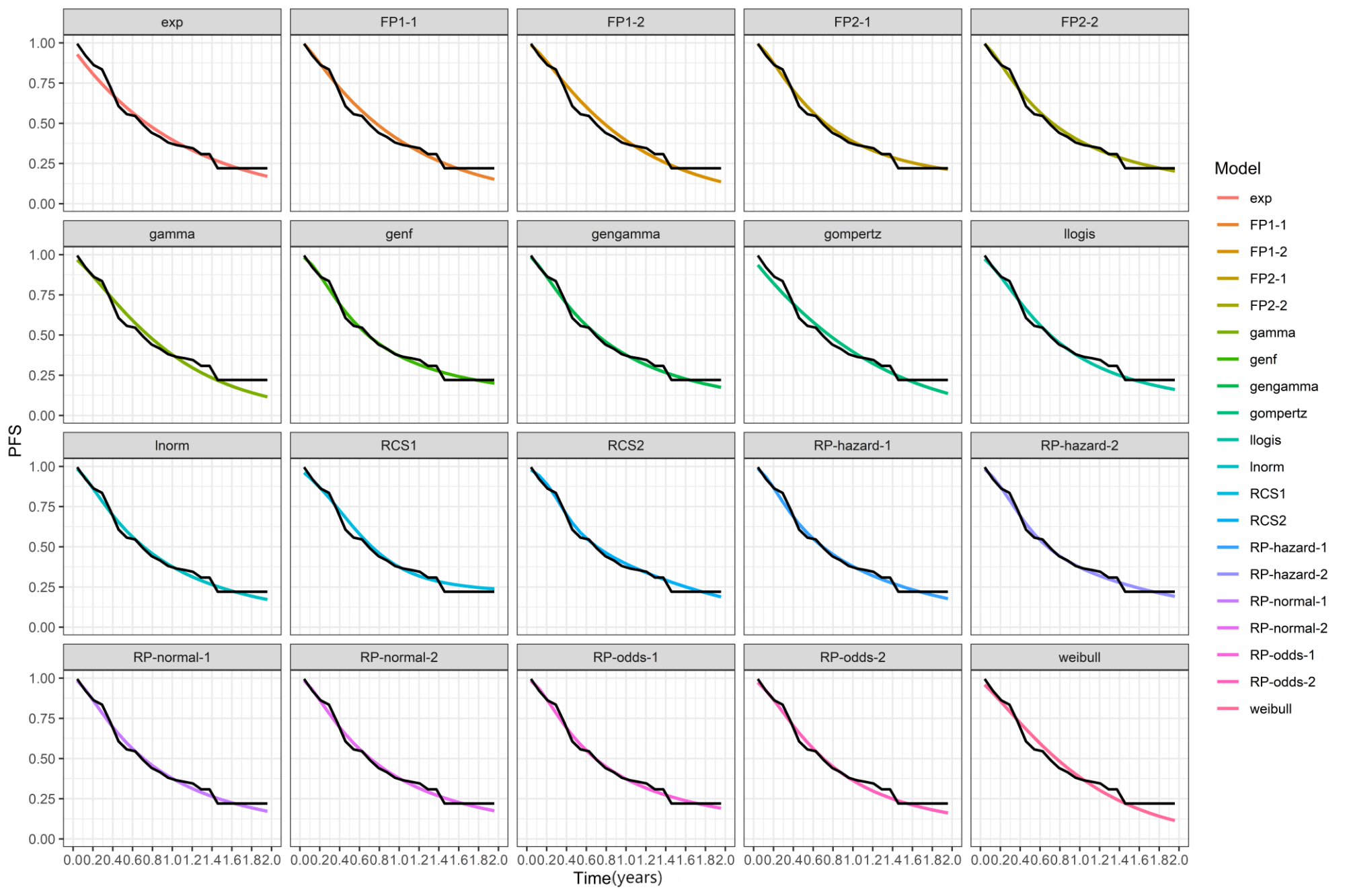


### 1.4 PFS of chemotherapy

| No | model | Knots/power | LnL | Params | AIC | Rank |
| --- | --- | --- | --- | --- | --- | --- |
| 1 | exp | / | -93.01 | 1 | 188.02 | 19 |
| 2 | weibull | / | -74.24 | 2 | 152.49 | 17 |
| 3 | gamma | / | -67.25 | 2 | 138.51 | 14 |
| 4 | lnorm | / | -59.47 | 2 | 122.95 | 4 |
| 5 | gompertz | / | -88.15 | 2 | 180.30 | 18 |
| 6 | llogistic | / | -60.37 | 2 | 124.73 | 6 |
| 7 | gengamma | / | -59.32 | 3 | 124.63 | 5 |
| 8 | genf | / |  |  |  |  |
| 9 | FP1_1 | -2 | -65.04 | 2 | 134.08 | 12 |
| 10 | FP1_2 | -1 | -68.05 | 2 | 140.10 | 15 |
| 11 | FP2_1 | -1;3 | -60.02 | 3 | 126.03 | 7 |
| 12 | FP2_2 | -0.5;3 | -60.47 | 3 | 126.93 | 9 |
| 13 | RCS1 | 1 | -64.00 | 3 | 133.99 | 11 |
| 14 | RCS2 | 2 | -63.99 | 4 | 135.98 | 13 |
| 15 | RP_hazard_1 | 5 | -57.60 | 7 | 129.20 | 10 |
| 16 | RP_hazard_2 | 2 | -59.17 | 4 | 126.35 | 8 |
| 17 | RP_odds_1 | 4 | -37.97 | 6 | 87.93 | 3 |
| 18 | RP_odds_2 | 5 | -65.09 | 7 | 144.17 | 16 |
| 19 | RP_lnorm_1 | 4 | -36.01 | 6 | 84.02 | 2 |
| ***20*** | ***RP_lnorm_2*** | ***3*** | ***-34.94*** | ***5*** | ***79.88*** | ***1*** |


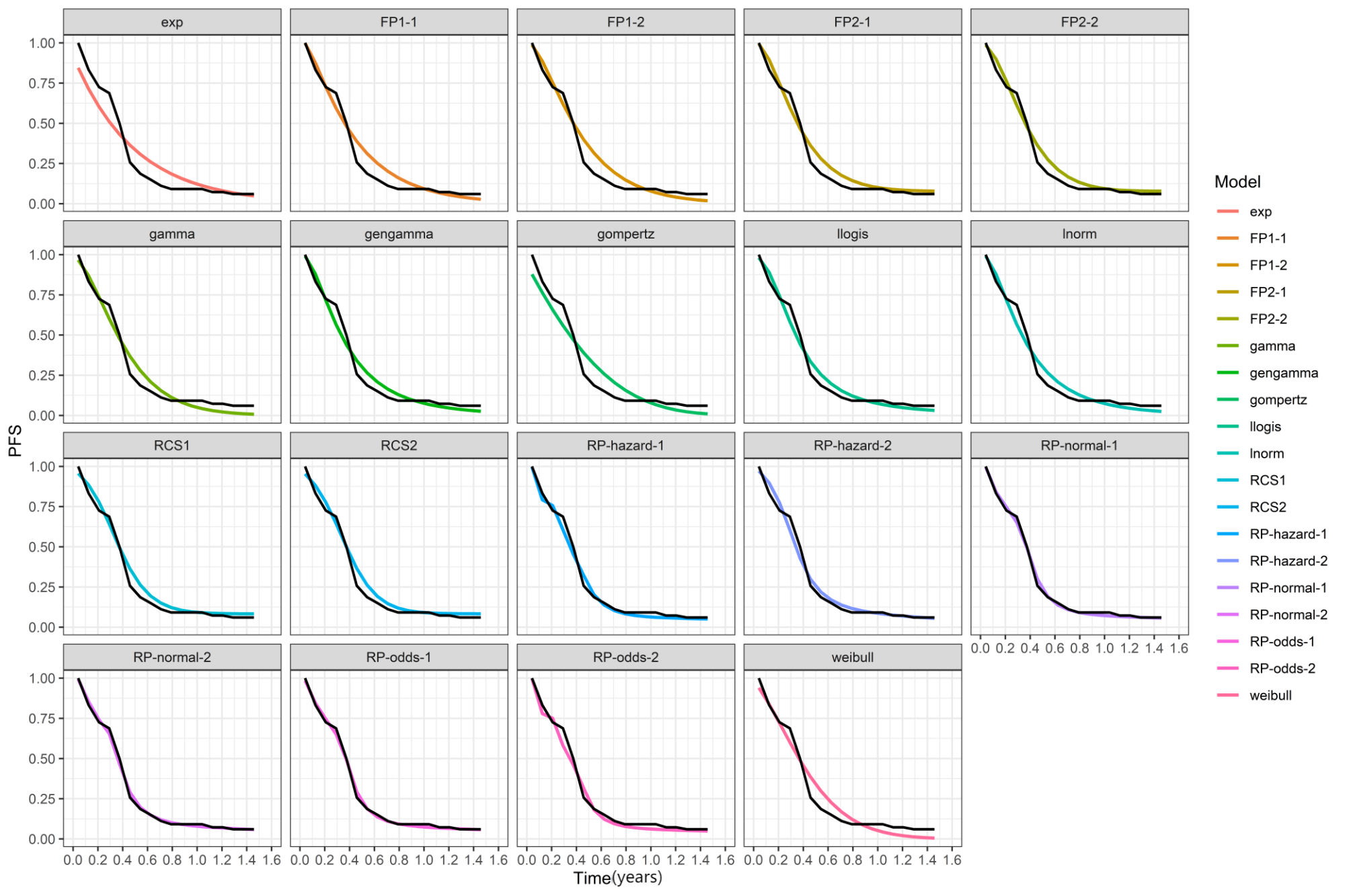


### 1.5 OS of Pembrolizumab

| No | Model | Knots/power | LnL | Params | AIC | Rank |
| --- | --- | --- | --- | --- | --- | --- |
| 1 | exp | / | -67.43 | 1 | 136.86 | 11 |
| 2 | weibull | / | -66.99 | 2 | 137.98 | 16 |
| 3 | gamma | / | -66.75 | 2 | 137.49 | 12 |
| 4 | lnorm | / | -68.80 | 2 | 141.60 | 20 |
| 5 | gompertz | / | -67.44 | 2 | 138.89 | 19 |
| 6 | llogis | / | -65.26 | 2 | 134.52 | 3 |
| 7 | gengamma | / | -65.99 | 3 | 137.98 | 15 |
| 8 | genf | / | -64.01 | 4 | 136.02 | 9 |
| 9 | FP1-1 | -0.5 | -66.80 | 2 | 137.60 | 13 |
| 10 | FP1-2 | -1 | -66.84 | 2 | 137.69 | 14 |
| 11 | FP2-1 | 0.5;1 | -64.86 | 3 | 135.72 | 7 |
| 12 | FP2-2 | 1;1 | -64.95 | 3 | 135.90 | 8 |
| 13 | RCS1 | 1 | -65.31 | 3 | 136.62 | 10 |
| ***14*** | ***RCS2*** | ***2*** | ***-63.18*** | ***4*** | ***134.36*** | ***1*** |
| 15 | RP-hazard-1 | 2 | -63.24 | 4 | 134.49 | 2 |
| 16 | RP-hazard-2 | 4 | -63.23 | 6 | 138.47 | 17 |
| 17 | RP-odds-1 | 2 | -63.57 | 4 | 135.14 | 5 |
| 18 | RP-odds-2 | 0 | -65.26 | 2 | 134.52 | 4 |
| 19 | RP-normal-1 | 2 | -63.71 | 4 | 135.41 | 6 |
| 20 | RP-normal-2 | 4 | -63.40 | 6 | 138.79 | 18 |


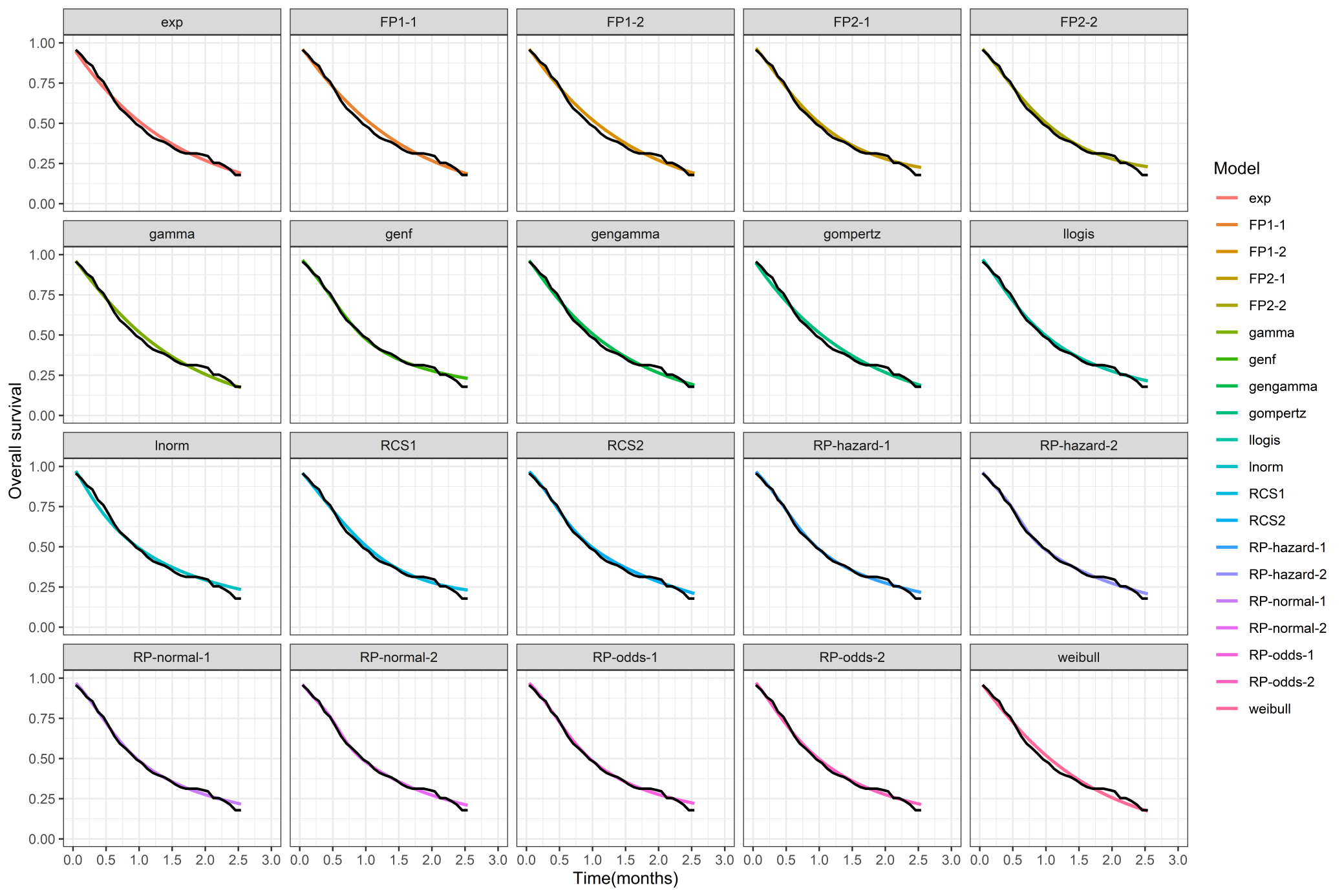


### 1.6 Explanation to the above figures and tables

Exp: exponential; logis: log-logistic; lnorm: log-normal; gengamma: generalized gamma; genf: generalized F; FP: fractional polynomial; RCS: restricted cubic spline models; RP: Royston-Parmar models;

### 1.7 Explanation of methodology used in fit and extrapolate survival outcomes

FP model, which was developed and put into use in the survival analysis by Royston and Altman(1-3). It was as an extension of polynomial functions that can be applied under the assumption of non-linearity of the hazard(4). FP could be divided into one-order FP and two-order FP with 8 optional powers {-2, -1, -0.5, 0, 0.5, 1, 2, 3}. Thus, there were eight one-order FP and 36 two-order FP. FP models are defined as follow(5):

Cubic spline model, also named RP model, was developed by Royston and Parmar. RP model was essentially a segmented cubic polynomial which needed to be continuously derivable at each segmented point(6). Model flexibility was based on the number of knots. A RP model could have a maximum of 5 internal knots and at least 2 knots (boundary knots). It was suggested that a maximum of three internal knots was usually sufficient, as larger values might lead to over-fitting. RP models are defined as follow (7):

$$\eta_{i}=\sum^{M+2} \gamma_{m}B_{im}$$

$B_{im}=(\log x-k_{m})_{+}^{3}-\lambda_{m}(\log x-k_{\min})_{+}^{3}-(1-\lambda_{m})(\log x-k_{\max})_{+}^{3}$

$\lambda_{m}=\frac{k_{\max}-k_{m}}{k_{\max}-k_{\min}}$

Where *B_im_* is referred to the basis function, $B_{i}=(B_{i0},B_{i1},\ldots,B_{iM})$. *k* refers to the knot and *M* refers to the number of intermediate nodes. When using RP models, we should consider three link functions: "hazard", "odds" and "normal".

Commonly, the boundary interval of cubic spline model was cubic polynomial, which could also be restricted to linear functions. The latter was called restricted spline model. RCS models are defined as follow (4,8):

$B_{im}=(x-k_{m})_{+}^{3}-\lambda_{m}(x-k_{\min})_{+}^{3}-(1-\lambda_{m})(x-k_{\max})_{+}^{3}$

$\lambda_{m}=\frac{k_{\max}-k_{m}}{k_{\max}-k_{\min}}$

Where *B_im_* refers to the basis function, $B_{i}=(B_{i0},B_{i1},\ldots,B_{iM})$. *k* is referred to the knot (generally one to five). Although RCS was flexible and could increase the complexity of the model by increasing the number of knots, it would lead to local over-fitting.

## The Replicated Kaplan-Meier PFS Curves


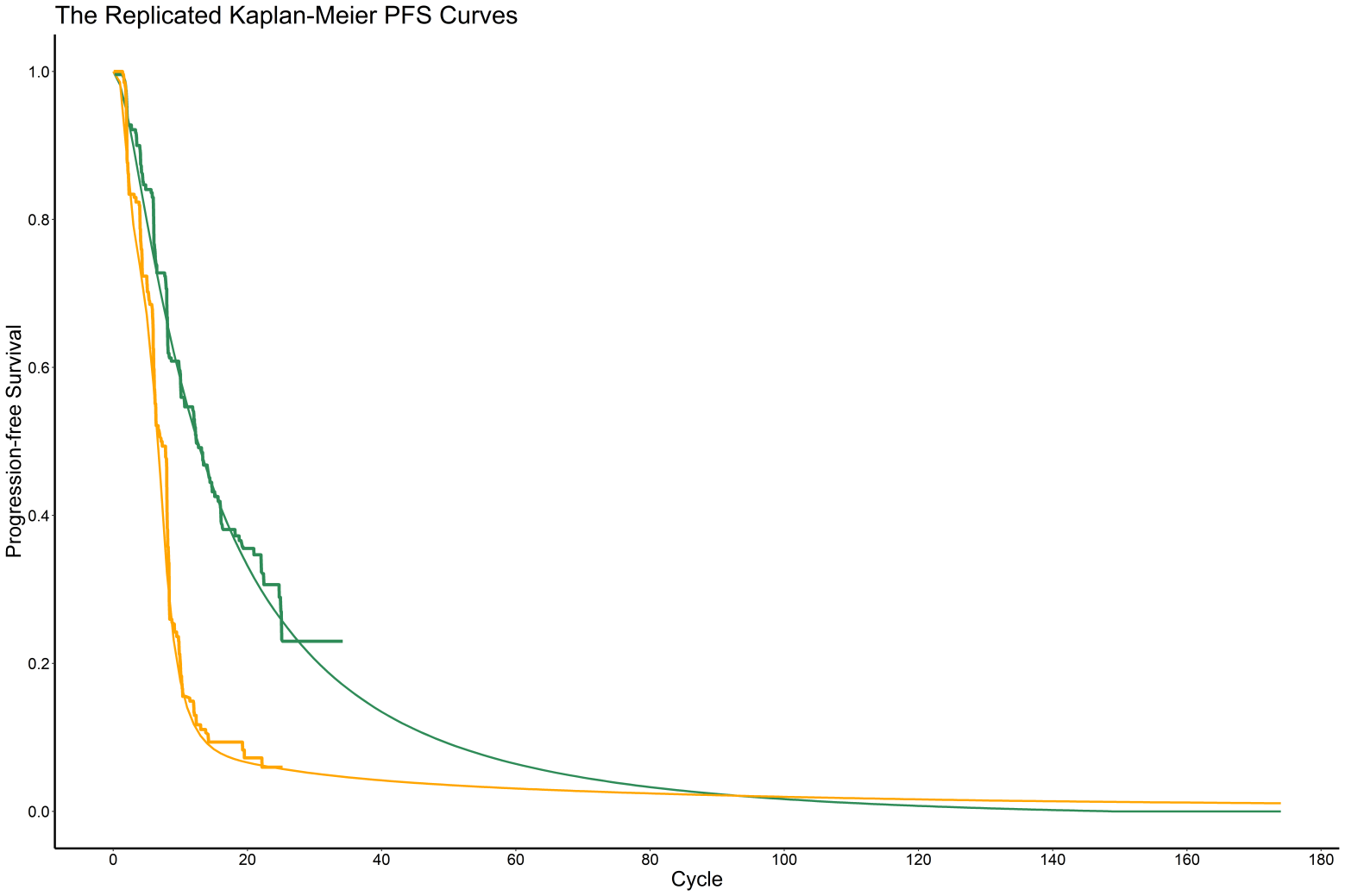


## The Replicated Kaplan-Meier OS Curves


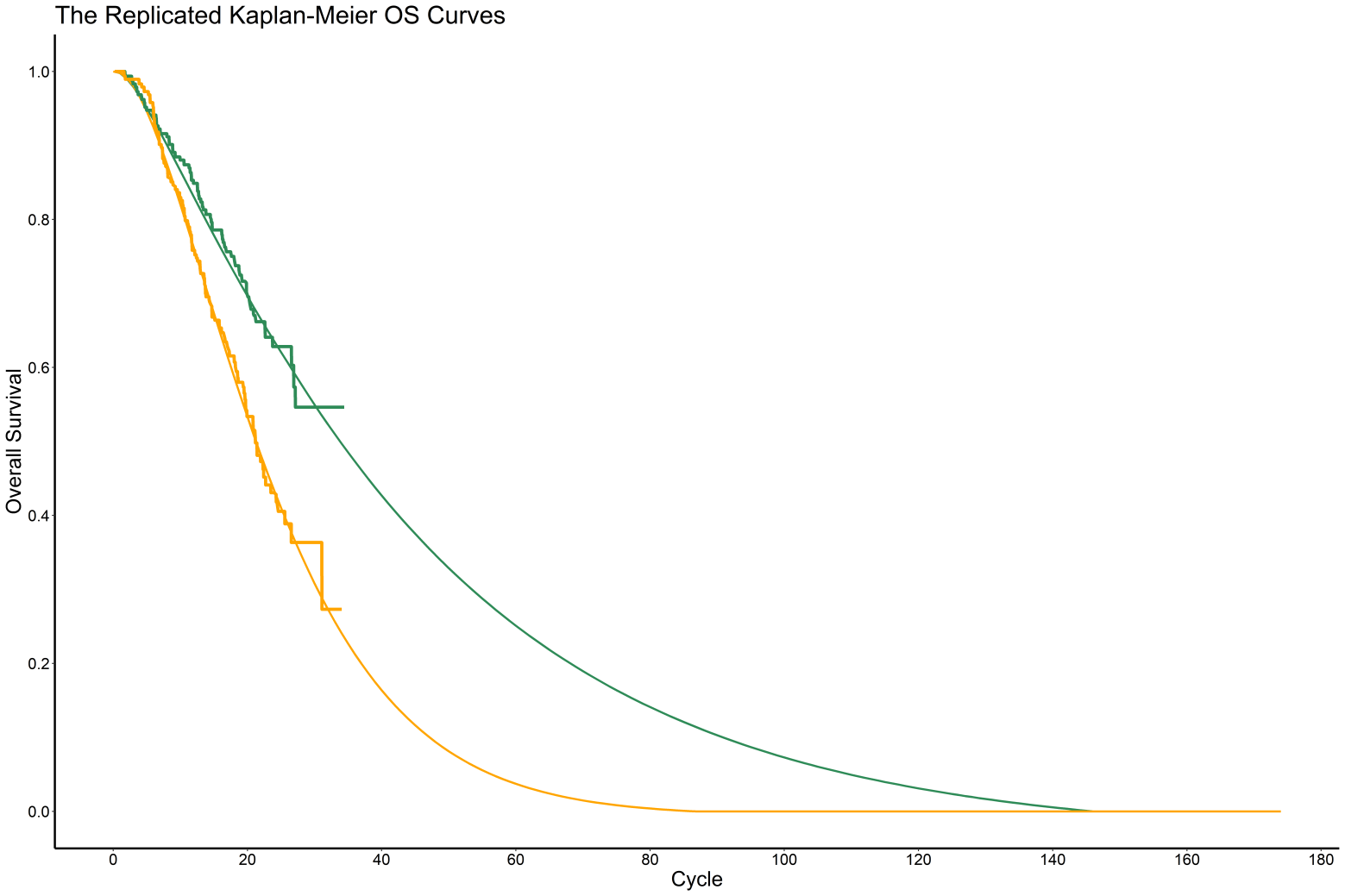


## CHEERS 2022 Checklist

| **Topic** | **No.** | **Item** | **Location where item is reported** |
| --- | --- | --- | --- |
| **Title** |  |  |  |
|  | 1 | Identify the study as an economic evaluation and specify the interventions being compared. | Page1 Line1-2 |
| **Abstract** |  |  |  |
|  | 2 | Provide a structured summary that highlights context, key methods, results, and alternative analyses. | Page1-2 Line16-40 |
| **Introduction** |  |  |  |
| **Background and objectives** | 3 | Give the context for the study, the study question, and its practical relevance for decision making in policy or practice. | Page3 Line51-82 |
| **Methods** |  |  |  |
| **Health economic analysis plan** | 4 | Indicate whether a health economic analysis plan was developed and where available. | Not applicable |
| **Study population** | 5 | Describe characteristics of the study population (such as age range, demographics, socioeconomic, or clinical characteristics). | Page3 Line88 |
| **Setting and location** | 6 | Provide relevant contextual information that may influence findings. | Page3-4 Line86-99,Line105-106 |
| **Comparators** | 7 | Describe the interventions or strategies being compared and why chosen. | Page3 Line88-92 |
| **Perspective** | 8 | State the perspective(s) adopted by the study and why chosen. | Page4 Line105-106 |
| **Time horizon** | 9 | State the time horizon for the study and why appropriate. | Page4 Line104-105 |
| **Discount rate** | 10 | Report the discount rate(s) and reason chosen. | Page4 Line108 |
| **Selection of outcomes** | 11 | Describe what outcomes were used as the measure(s) of benefit(s) and harm(s). | Page4 Line106-107 |
| **Measurement of outcomes** | 12 | Describe how outcomes used to capture benefit(s) and harm(s) were measured. | Page4 Line106-107 |
| **Valuation of outcomes** | 13 | Describe the population and methods used to measure and value outcomes. | Page4 Line106-107 |
| **Measurement and valuation of resources and costs** | 14 | Describe how costs were valued. | Page5 Line127-140 |
| **Currency, price date, and conversion** | 15 | Report the dates of the estimated resource quantities and unit costs, plus the currency and year of conversion. | Page4 Line131 |
| **Rationale and description of model** | 16 | If modelling is used, describe in detail and why used. Report if the model is publicly available and where it can be accessed. | Page4 Line100-112 |
| **Analytics and assumptions** | 17 | Describe any methods for analysing or statistically transforming data, any extrapolation methods, and approaches for validating any model used. | Page5 Line114-125 |
| **Characterising heterogeneity** | 18 | Describe any methods used for estimating how the results of the study vary for subgroups. | Page5 Line169-173 |
| **Characterising distributional effects** | 19 | Describe how impacts are distributed across different individuals or adjustments made to reflect priority populations. | Page5 Line158-167 |
| **Characterising uncertainty** | 20 | Describe methods to characterise any sources of uncertainty in the analysis. | Page5 Line150-173 |
| **Approach to engagement with patients and others affected by the study** | 21 | Describe any approaches to engage patients or service recipients, the general public, communities, or stakeholders (such as clinicians or payers) in the design of the study. | Not applicable |
| **Results** |  |  |  |
| **Study parameters** | 22 | Report all analytic inputs (such as values, ranges, references) including uncertainty or distributional assumptions. | Table1 |
| **Summary of main results** | 23 | Report the mean values for the main categories of costs and outcomes of interest and summarise them in the most appropriate overall measure. | Page5-6 Line177-183 |
| **Effect of uncertainty** | 24 | Describe how uncertainty about analytic judgments, inputs, or projections affect findings. Report the effect of choice of discount rate and time horizon, if applicable. | Page6 Line185-196 |
| **Effect of engagement with patients and others affected by the study** | 25 | Report on any difference patient/service recipient, general public, community, or stakeholder involvement made to the approach or findings of the study | Page5-6 Line199-216 |
| **Discussion** |  |  |  |
| **Study findings, limitations, generalisability, and current knowledge** | 26 | Report key findings, limitations, ethical or equity considerations not captured, and how these could affect patients, policy, or practice. | Page6-8 Line219-284 |
| **Other relevant information** |  |  |  |
| **Source of funding** | 27 | Describe how the study was funded and any role of the funder in the identification, design, conduct, and reporting of the analysis | Page8 Line294-295 |
| **Conflicts of interest** | 28 | Report authors conflicts of interest according to journal or International Committee of Medical Journal Editors requirements. | Page8 Line298-300 |

*From:* Husereau D, Drummond M, Augustovski F, et al. Consolidated Health Economic Evaluation Reporting Standards 2022 (CHEERS 2022) Explanation and Elaboration: A Report of the ISPOR CHEERS II Good Practices Task Force. Value Health 2022;25. <doi:10.1016/j.jval.2021.10.008>

References:

1. Royston P, Sauerbrei W. A new approach to modelling interactions between treatment and continuous covariates in clinical trials by using fractional polynomials. *Stat Med*. (2004) 23(16):2509-2525. doi:10.1002/sim.1815

2. Royston P, Sauerbrei W. Building multivariable regression models with continuous covariates in clinical epidemiology--with an emphasis on fractional polynomials. *Methods Inf Med*. (2005) 44(4):561-571.

3. Royston P, Altman DG. Regression Using Fractional Polynomials of Continuous Covariates: Parsimonious Parametric Modelling. *Insurance Mathematics and Economics*. (1995) 16(2):165-166.

4. Kearns B, Stevenson MD, Triantafyllopoulos K, Manca A. Generalized Linear Models for Flexible Parametric Modeling of the Hazard Function. *Med Decis Making*. (2019) 39(7):867-878. doi:10.1177/0272989X19873661

5. Jansen JP. Network meta-analysis of survival data with fractional polynomials. *Bmc Med Res Methodol*. (2011) 11:61. doi:10.1186/1471-2288-11-61

6. Lambert PC, Royston P. Flexible parametric survival analysis using Stata :beyond the Cox model. (2011). College Station, TX: Stata Press.

7. Latimer NR. Survival analysis for economic evaluations alongside clinical trials--extrapolation with patient-level data: inconsistencies, limitations, and a practical guide. *Med Decis Making*. (2013) 33(6):743-754. doi:10.1177/0272989X12472398

8. Rutherford MJ, Crowther MJ, Lambert PC. The use of restricted cubic splines to approximate complex hazard functions in the analysis of time-to-event data: a simulation study. *J Stat Comput Sim*. (2015) 85(4-6):777-793.
